# Expanding Robotic-Assisted Surgery in Gynecology Using the Potential of an Advanced Robotic System

**DOI:** 10.3390/medicina60010053

**Published:** 2023-12-27

**Authors:** Ibrahim Alkatout, Odile O’Sullivan, Göntje Peters, Nicolai Maass

**Affiliations:** 1 Department of Gynecology and Obstetrics, University Hospitals Schleswig-Holstein, Campus Kiel, D-24105 Kiel, Germany; 2 Distalmotion SA, Route de la Corniche 3b, 1066 Epalinges, Switzerland; odile.osullivan@distalmotion.com

**Keywords:** gynecological surgical procedures, minimally invasive surgical procedures, robotic-assisted surgery, Dexter System, laparoscopy

## Abstract

Minimally invasive surgery (MIS) in gynecology was introduced to achieve the same surgical objectives as traditional open surgery while minimizing trauma to surrounding tissues, reducing pain, accelerating recovery, and improving overall patient outcomes. Minimally invasive approaches, such as laparoscopic and robotic-assisted surgeries, have become the standard for many gynecological procedures. In this review, we aim to summarize the advantages and main limitations to a broader adoption of robotic-assisted surgery compared to laparoscopic surgeries in gynecology. We present a new surgical system, the Dexter Robotic System™ (Distalmotion, Switzerland), that facilitates the transition from laparoscopy expertise to robotic-assisted surgery.

## 1. Introduction—Trends in Minimally Invasive Surgery in Gynecology

Laparoscopic surgery (LAP) evolved gradually in the past century, ultimately achieving successful implementation in the late 1980s [1]. Despite its initial slow adoption, LAP has become a standard approach in the past four decades [2]. The advantages of LAP compared to open surgery are well established. Utilizing small incisions (ranging from 5 to 12 mm) and specialized surgical instruments, LAP minimizes damage to surrounding tissues, resulting in reduced pain, decreased blood loss, fewer postoperative complications, significantly shorter hospital stays, faster recovery, and lower morbidity when compared to traditional open surgery [3,4,5].

Robotic-Assisted Surgery (RAS) has provided further advantages to surgeons over laparoscopic techniques. Ever since the first Food and Drug Administration (FDA) approval in 2000, RAS applications have expanded significantly. Nonetheless, despite over twenty years since its inception, the adoption of RAS still shows discrepancies across various surgical fields. Urology stands out as a field where RAS has been at the forefront of this adoption process, fundamentally transforming the landscape of minimally invasive surgery (MIS) within the last decade [6]. In urology, the shift from open surgery to MIS faced challenges due to the steep learning curve associated with LAP. This transition was accelerated with the approval of RAS, which significantly shortens the learning curve by nearly tenfold [7]. A recent study [8] has also shown that during the training in general surgery procedures such as hepaticojejunostomy and gastrojejunostomy, residents rated more nervousness and anxiety for laparoscopy compared to robotic- assisted surgery. Their technical performance was actually superior in robotic drills compared to laparoscopic drills [8]. On the other hand, the adoption of RAS in gynecological surgery is progressing at a slower pace. Today, RAS has been performed in numerous benign [9,10] and malignant [11] conditions such as hysterectomy, oophorectomy, salpingectomy, myomectomy, ovarian cystectomy, lymphadenectomy, endometriosis surgery, sacrocolpopexy, and pelvic exenteration, with a tendency to keep expanding to new indications. Hysterectomies, as the most frequently performed major gynecological surgical procedures, have been transitioning to the RAS approach at a relatively rapid pace [12]. RAS hysterectomies were proven to be noninferior to conventional LAP hysterectomies [13,14]. Due to shorter hospital stays for RAS [13], intraoperative complication rates and, consequently, the conversion rate were either unchanged [15,16,17,18] or reduced in RAS [4,19], although one study reports more postoperative pain after RAS relative to LAP [16].

The benefits of MIS have led to a notable trend shift in gynecological surgery, as evidenced by studies reporting significant reductions in open surgery rates [20,21,22,23]. Numerous published reviews on robotics in gynecological surgery highlight a strong interest within the surgical community to develop awareness and accelerate the adoption of more advanced technologies [9,10,11,24]. Despite recommendations from international guidelines [25,26], a few factors continue to hinder the complete replacement of traditional open surgery with MIS. Gynecologist surgeons can perform almost all surgeries using conventional laparoscopy and don’t seem to require further development of advanced technological systems. Convincing them to increase the practice of RAS remains difficult among the community. Is this due to technological limitations or access or both? We propose to discuss the advantages and limitations of RAS when compared to laparoscopic surgeries. Some of these limitations may be addressed in a new robotic platform recently introduced on the European market and indicated for gynecology surgeries, the Dexter Robotic System™.

## 2. Advantages of RAS for Surgeons

The advantages of robotic assistance for surgeons are clearly documented. Laparoscopic instruments only allow limited degrees of freedom of movement, as they cannot be deflected at the tip. Robotic instruments offer a greater range of motion compared to traditional laparoscopic instruments. This enhanced dexterity is particularly advantageous in intricated surgical steps such as suturing, fine dissection, or delicate tissue manipulation. Robotic instruments can also replicate movements of a human wrist, enabling greater flexibility in maneuvering with confined spaces. In addition, robotic systems filter out hand tremors, providing steady and precise movements, which is especially useful for tasks requiring high precision. The camera is stable and can be controlled on demand by the surgeon. This leads to a higher level of concentration on the area of interest if no communication with the assistant is needed and if there is no compromise on the stability of the picture. A study by Advincula and Reynolds was one of the first feasibility studies to suggest a potential role for robotics in overcoming the technical limitations of conventional laparoscopy for hysterectomy cases with an obliterated anterior cul-de-sac [27].

Surgeons operate robotic systems from a comfortable console, reducing physical strain and fatigue during long procedures. This ergonomic advantage can lead to improved surgical precision. A recent systematic review and meta-analysis revealed that 82% of gynecological surgeons performing laparoscopic procedures experience musculoskeletal symptoms [28]. Lengthy or complex procedures such as sacrocolpopexy, for example, may be more likely to cause such symptoms. Compared to LAP, there is less occurrence of work-related musculoskeletal disorders in robotic surgeons thanks to the seated, ergonomic position [29]. This has a potential effect on the length of the surgeon’s career [30].

In some instances, the use of robotics may confer certain advantages, such as autonomy when limited or no assistance is available, with improved or similar perioperative outcomes compared with other surgical approaches [31].

While the advantages that robotic assistance bring to the surgeon are undisputed, the impact on the surgical performance and benefits for patients remains a topic of debate in the literature.

## 3. Equivalence of RAS and LAP in Patient Outcomes

Numerous studies have demonstrated that both LAP and RAS are effective in achieving surgical goals. Table 1 summarizes the main study results of the largest randomized controlled trials and meta-analyses we retrieved from the literature. Studies have indicated that the rates of postoperative complications, such as infections, bleeding, and wound complications, are generally comparable between laparoscopy and RAS [32]. The choice of approach does not seem to significantly affect the overall complication profile. Patients undergoing either LAP or RAS tend to experience shorter hospital stays, reduced pain, and faster recovery times compared to open surgery. Long-term satisfaction of patients after LAP and RAS were found to be similar [33], and women are generally satisfied with their decision to undergo robotic surgery [34]. However, a recent meta-analysis on the return to normal activities, satisfaction, and quality of life has found inconclusive results from the three RCTs available in the literature [35].

Overall, the meta-analyses also reveal that while data to support the feasibility for various gynecological procedures exist, there is a limited number of reliable, high-quality comparative studies demonstrating the superiority of RAS over LAP [10,11]. When limited to RCT, the robotic approach was not found to significantly improve perioperative outcomes when compared to LAP [32,36]. Prospective studies are also rare, and most of them are small observational trials which are often underpowered. Larger retrospective meta-analyses can provide insights into the available evidence, but they are also prone to the selection bias (often reporting on specific patient populations) as well as the information bias (relying on the accuracy and completeness of medical records and surveys) [4,13,15,36]. Limitations in the number of active robotic surgeons in gynecology, their experience, and preference for surgical approach can hinder adequate comparison between different available surgical approaches. Several studies have small sample sizes, short follow-up periods, or feature different designs and robotic system used, which can impact the strength of the evidence [37]. Furthermore, some of these studies are potentially biased by the inclusion of early robot adopters, which can affect results such as operating times, complication rates, and estimated blood loss [11,15,37]. This makes it challenging to draw robust conclusions and increases the risk of inappropriate pooling of data. The meta-analysis of Lenfant et al. [36] has revealed significant heterogeneity in the pooled data from RCTs, prospective studies, and retrospective studies versus analyses and differences in the subgroup analyses by study type.

Taking into consideration the presently available studies and bearing in mind the current state of the art in surgical instrumentation and surgical techniques, currently RAS in gynecology is not necessarily identified as an exclusive alternative to laparoscopy, but rather as the complementary next step in the process of technological development [11,13]. Whether it is only a stopover type of surgery or it is a next-level surgery that will be enhanced with each new generation cannot be answered today. This is dependent on many items such as general technical development, global digitalization, health politics, and others.

## 4. Equivalence of RAS and LAP in Technology Adoption Challenges (Training and Learning Curve)

A frequent observation in comparative studies is that operating time (OT) is unchanged between the two approaches and occasionally reported to be longer for RAS [13,17,41], although there is evidence to the contrary as well [19] (Table 1).

Appropriate training on robotic devices is necessary to ensure patient safety and the appropriate use of technology. The learning curve can be steep, and not all surgeons have access to adequate training opportunities. Both the American College of Obstetricians and Gynecologists (ACOG) and the American Association of Gynecologic Laparoscopists (AAGL) have release statements recommending rigorous training and credentialing standards, minimum case numbers, proctoring, and peer case review [25,26]. According to the SERGS recommendation [42], the new trainees are to be mentored/proctored by an experienced trainer for 10 cases [43].

One important factor in reaching optimal surgical performance is the learning curve. Advanced endoscopic operations are not easy to learn and master. Even with years of experience, LAP introduces inherent drawbacks that can affect the surgeon’s performance. These drawbacks lie in the loss of depth perception when using a 2D endoscope, an unstable video camera when it is held manually, limited dexterity, counterintuitive and limited movement of LAP surgical instruments (due to the enforced fixation by the trocars and no deflection at the tip), the fulcrum effect, and very poor ergonomics for the surgeon and their assistants over extensive operation time [44,45]. Innovation in endoscopic systems such as high-resolution 3D cameras enabling better exposure and anatomical mapping of the operating space has recently further improved surgical performance during LAP [46,47,48]. Surgeons can now navigate anatomical structures with greater clarity, leading to improved outcomes and reduced operation times. Although standalone 3D endoscopic cameras are available, their adoption in the OR of laparoscopic surgeons remains rare. The combination of improved 3D visualization and instrument control without counterintuitive hand movements (as is the case with LAP) should enable a faster surgical learning curve on a robot compared to LAP [49,50]. The linear regression of operation times shows a significant reduction after the first 30 cases of robotically assisted hysterectomies [41]. However, they do not always compensate for the lack of haptic feedback, which some surgeons rely on for precise adjustment and judgement during their laparoscopic procedures. This transition requires specific training and neuro-adaptation skills acquisition. Finally, regular practice and the use of robotic systems are necessary to maintain surgical skills [51]. Surgeons who use the system infrequently might struggle to maintain proficiency. Additionally, nursing staff must also be trained in the use of the robot system. MIS training and the LAP experience of medical personnel already help in smoothing the learning curve for RAS [52].

## 5. Limitations of RAS and Advantages of LAP

The implementation of a robotic surgery program, while offering numerous benefits, can however be limited by certain practical factors, such as the large footprint of the robot and the sterilization processes involved. Robotic surgical systems such as the da Vinci Surgical System typically occupy a significant amount of space within the operating room [53]. This large footprint can limit the flexibility of arranging surgical equipment and personnel at the bedside during procedures to avoid collisions. Smaller operating rooms may find it challenging to accommodate large robots, potentially leading to logistical issues and reduced maneuverability for the OR team. The robot’s size can obstruct access to the patient or surgical site in some cases. Retrofitting an operating room to accommodate a robotic system may require additional investments in infrastructure, including modifications to the OR layout, electrical system, and space allocation. These modifications can be costly and time-consuming. This is why we see an emergence of new robots proposing various modalities and separate carts to distribute around the patient bed [54]. The majority of currently available robotic systems on the market, including the da Vinci Surgical System (Intuitive Surgical, Inc., Sunnyvale, CA, USA), Versius Surgical Robotic System (CMR Surgical, Cambridge, UK) [55], and HUGO RAS System (Medtronic, Minneapolis, MN, USA) feature four-arm setups. Whether the robotic arms are integrated in one unit, or carried by four separate ones, their total volume adds to the bulkiness of the system in the OR as well as in the workspace above and around the patient, thus leaving little room for the assisting surgical staff and often enforcing on them ergonomically unfavorable working positions, accelerating their fatigue and hindering optimal patient access [54]. In LAP, the surgeon stands at the patient’s bedside at all times, surrounded by his surgical team. He directly controls the instruments, providing a tactile sensation (haptic feedback) and direct manipulation of the tools.

Some limitations of the da Vinci closed surgeon console have been identified, such as the difficulty for surgeons to interact directly with the surgical team [56]. This can be limiting for making critical decisions that require immediate communication with the surgical team. The study conducted by AAGL on experienced robotic surgeons in gynecology also reported that more than half of participants still experience physical symptoms primarily associated with confidence in managing ergonomics settings at the console [56]. The newer robotic platforms are making changes to the surgeon console design, allowing the surgeon to sit or stand in an open console, facilitating visual exchange with the OR staff.

While robotic systems offer enhanced dexterity and precision, their complexity can lead to technical challenges during surgery. Malfunctions or technical issues can disrupt procedures. Furthermore, robotic systems permit the surgeon to perform endoscopic surgery only if the ports are positioned appropriately and no arm collides with other arms [57]. The robot’s size can obstruct access to the patient or the surgical site, making it less suitable for procedures that require multiple angles or involve complex positioning of the patient. The workspace reached by the robot instrumentation may limit the freedom of port placement and require multiple trocar placements, leading to more incision scars than in LAP. Some patient morphology or anatomy may hinder the possibility of performing RAS due to challenging port placement or robot docking. Urologists have been early adopters of robotic surgery because the depth of the pelvis makes it harder to access and also because the structures that are significant in the field are very small, with fewer surrounding structures. In contrast, gynecological procedures often involve complex pelvic anatomy, including organs such as the uterus, ovaries, fallopian tubes, and surrounding structures. The confined pelvic space can make access more challenging during robotic surgery and may require multiple entry points within the pelvic region, including the natural orifices. When electromechanical morcellation is needed for example, it can be performed more easily through the laparoscopic approach because the location of trocars in robotically assisted surgery (in a straight line at the level of the umbilicus or higher) is unsuitable for electromechanical morcellation.

Proper cleaning and reprocessing of robotic instruments that are not single use require dedicated resources and processes to ensure patient safety. Reprocessing robotic instruments is generally more complex and can involve higher consumable costs, specialized training, and maintenance requirements compared to laparoscopic instruments. These procedures come with associated costs, are time-consuming, and potentially impact OR efficiency and patient scheduling. However, reusable robotic instruments reduce most impacts on the environment except water use [58].

Each hospital may establish its own policies and criteria for granting privileges to use robotic systems. These policies may consider factors such as surgeon training and experience, case volumes, and patients’ outcomes when determining which specialties gain access to the technology [24]. Robotic platforms are typically shared across multiple disciplines within institutions. The hospital administration and payers assess procedures costs and associated reimbursements to allocate robotic access among different specialties. Gynecology procedures, especially benign cases, often receive lower reimbursement coverage when compared to urological or complex general surgery interventions [59], resulting in limited access to the robot. LAP procedures are very well established and cost-efficient. On the public side however, a 2016 study showed that patients undergoing surgery in a hospital in a competitive regional market were more likely to undergo a robotically assisted procedure [60]. Patients often see the adoption of new technology as an indicator of high-quality care. Additionally, advanced technology acquisition may help hospitals recruit surgeons who are interested in using robotic surgical systems; hospital decisions to purchase robotic machines are mainly driven by this market pressure [61].

Given the inherent costs of RAS, the majority of robotic cases in gynecology were performed for malignant indications initially. Today, benign conditions are treated robotically as well, so RAS approaches have been described for numerous procedures, including hysterectomy, myomectomy, sacrocolpopexy, endometriosis surgery, and a few others. RAS surgery was found to be associated with increased incremental disposable costs per case and total hospital charges when compared to LAP [62]. Costs are indirectly influenced by the OR team workflow, postoperative processes to expedite discharge, and converting surgery to the ambulatory setting. It is still highly argued that more evidence is needed to develop evidenced-based practices for cost containment in robotic surgery [63]. However, robotic platforms are multi-indication tools, not therapies, and should be evaluated in this context. Individual procedure-by-procedure assessments may not be appropriate, and raising the awareness of more adequate health technology assessments is now acknowledged by an international consensus panel [64].

A natural consequence of technological advancement in the market is the emergence of newer robotic surgical platforms. As they seek to compete with the established da Vinci Surgical Systems, several important areas are being targeted with new innovations: surgeon ergonomics, visualization, the incorporation of haptic feedback, and reductions in the overall footprint, which includes making the robotic platform itself more compact as well as decreasing the size and number of incisions [24,54].

The expansion of the competition landscape in RAS is driving the technological evolution of robots. These advancements hold the promise of delivering innovative solutions for indications that were previously unaccounted for, reducing costs, and expanding the range of gynecological procedures suitable for RAS. Until now, hospitals have either had robotically assisted surgery capabilities or they have not. The absence of a hybrid laparoscopic surgical system left no middle ground. However, the landscape is changing, and we are now suggesting Dexter that might fill the gap.

## 6. On-Demand Robotic Assistance

**Figure 1:**
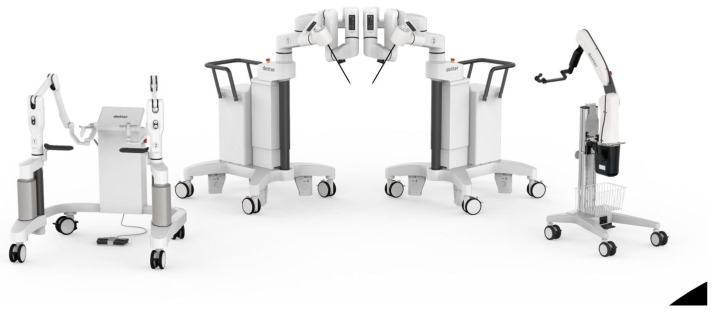
The Dexter robotic system, consisting of an open, sterile surgeon console, two patient carts with robotic arms, and an endoscope cart.

The Dexter Robotic System™ (Distalmotion SA, Epalinges, Switzerland) represents an alternative to traditional robotic systems. Dexter is a modular robotic platform comprising an open, sterile, and ergonomically designed surgeon console accompanied by two patient carts, each equipped with a robotic arm. Additionally, it includes a robotic endoscope arm capable of accommodating any 3D endoscopic system, all controllable from the surgeon console (Figure 1). The console is equipped with endoscope and clutch pedals, providing the surgeon with seamless control over the instrument maneuvering and the field of view adjustments. Dexter is intentionally conceived as an open platform for imaging systems, allowing the surgeon to select which 3D/fluorescence imaging system they wish to use. This offers the flexibility to incorporate cutting-edge imaging systems with routine updates or continue utilizing existing 3D/fluorescence imaging systems already in place in the OR, integrated into the conventional LAP setup. This open platform approach is equally applicable for energy devices, which now have become indispensable equipment in every OR. Table 2 summarizes the principal characteristics of Dexter.

Dexter seamlessly integrates into any operating room setting. Its mobile design and modest weight enable transportation between rooms, facilitating its shared utilization across multiple OR and surgical departments. This not only enhances procedural efficiency but also ensures optimal utilization of the robot within the hospital. In addition, the whole system has a compact form factor, enabling easy storage and freeing up space in the OR when the robot is not in use.

**Figure 2:**
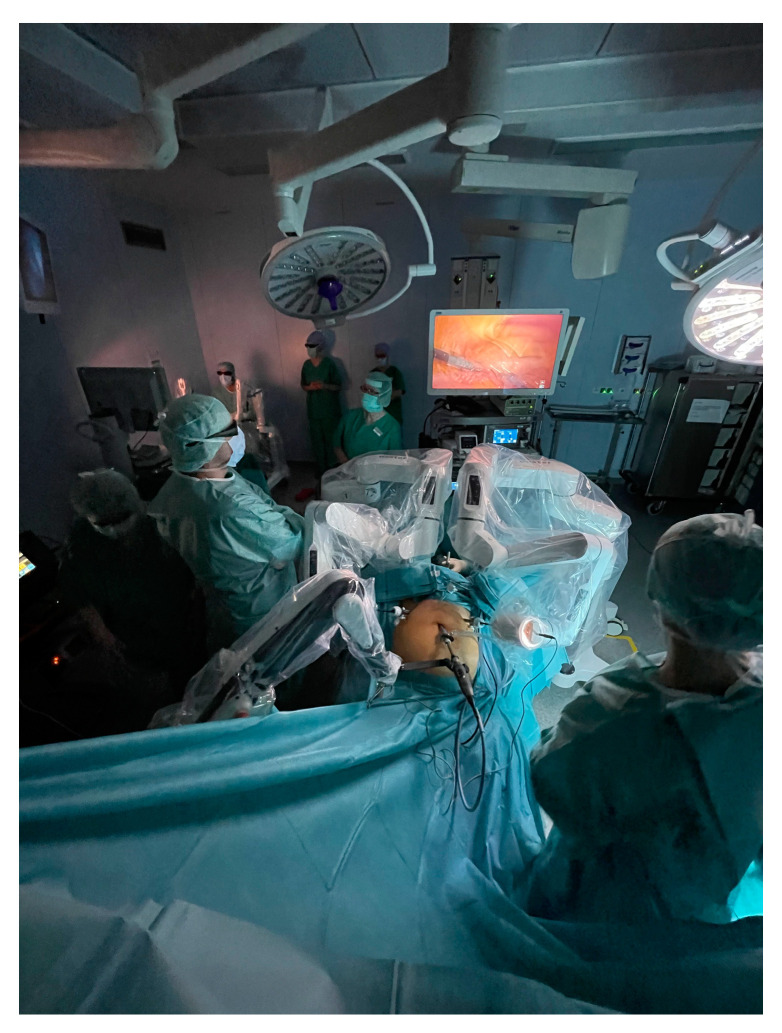
Full view of the operating room during a Dexter robotic system surgery. From the sterile surgeon console (background upper left quadrant of the photo), the surgeon is controlling the two robotic arms. The open space and large 3D 4K screen allow the assistants and residents to observe the case in real time. The assistant surgeon and sterile nurse both have a sufficient working area at the patient’s bedside.

One of Dexter’s innovative features is its ability to facilitate a smooth transition from LAP surgery to RAS. Dexter’s concept of the on-demand approach [65,66,67] implies utilizing LAP in scenarios where speed and special instruments are desired and employing RAS for parts of the surgical procedure where enhanced ergonomics, precise instrument control, and maneuvering in confined spaces, such as suturing and dissection, are required. By preserving the conventional LAP port placements [68], the surgeon can keep using their preferred approach based on the patient’s anatomy and the required technique. For this purpose, the Dexter robotic system is designed for swift modality changes between RAS and LAP, with its unique feature, the LAP mode, enabling each robot arm to be folded away from the surgical field within less than 30 s (unpublished data) with a simple button push, all without the need to undock them. This flexibility should facilitate speed, ease of maneuvering, confidence in the approach, and the use of well-established surgical instruments that the surgeon has mastered and that are readily available in the hospital’s inventory. Moreover, Dexter’s patient carts are designed with consideration for the existing walking paths and roles of the surgical team, similar to those in LAP procedures. This design ensures sufficient working space at the patient’s bedside (Figure 2), avoiding obstruction of assisting surgical staff, which is often the case with conventional robotic platforms [69,70].

Speaking and being understood in the operating room is essential for facilitating cooperation between the surgeon and his team. The Dexter open console enables immediate open communication and interaction with all the surgical staff present in the room. The compact setup of the robotic arms leaves a wide operating space in the sterile area at the patient’s bedside for the assisting surgical team. The Dexter’s sterile environment allows proximity between the surgical team, enabling interaction, easy observation, training, and support between the surgeon and trainees both at the patient bedside and at the surgeon console without requiring a second surgeon console (Figure 2). Finally, in emergency situations, the surgeon’s sterility remains uncompromised throughout the operation, enabling them to swiftly respond at the patient’s bedside, which not only can mean a safer procedure, but can also reduce the mental workload on the surgical assistant, who is otherwise alone at the patient’s bedside in cases where the surgeon needs to scrub in amidst conventional RAS.

**Figure 3:**
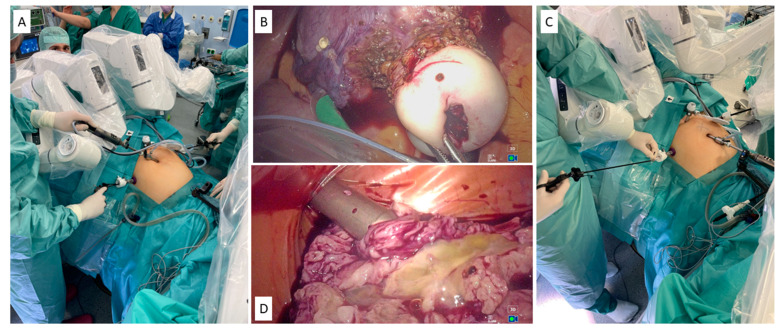
(**A**) The robot arms have been put in LAP mode. The surgeon is at the patient bedside operating using the same trocar placement that is used during the robotic mode when performing upper quadrant procedure steps. (**B**) Operating in LAP mode, the surgeon is taking the uterus from the upper quadrant in the pelvis to be put in the morcellation bag using laparoscopic tools on existing trocars. (**C**) The surgeon operating in LAP mode, inserting the morcellation bag and then the power morcellator via the lower left working trocar that was used during the robotically assisted dissection. (**D**) In-bag morcellation performed in laparoscopic mode during a robotically assisted surgery procedure.

In general, certain procedural steps in gynecological surgeries either require a well-trained surgical assistant, which is becoming increasingly rare in daily theatre practice, or the active participation of the surgeon. This can lead to the surgeon scrubbing in again, causing delays in the procedure and requiring the surgical team to adapt to the new situation. Those situations occur, for example, during myoma resections after enucleation and uterine reconstruction, when the myoma needs to be morcellated. Morcellators are available only in conventional laparoscopy. Similar situations occur if a subtotal hysterectomy is performed and the uterine corpus is resected through morcellation [71]. Another reason could be the change in the surgical field, from the lower abdomen to upper abdomen, to perform minor surgical steps such as adhesiolysis or biopsy. As a result, re-docking the robotic system is much more time consuming compared to simple laparoscopy, impacting the continuity of surgery in the initial field. The LAP mode feature of the Dexter System grants the main operating surgeon complete open access to swiftly perform critical steps, enabling the surgeon to seamlessly transition to laparoscopic mode, facilitating on-demand robotically assisted surgery for the efficient and precise execution of crucial surgical maneuvers such as uterus manipulation and morcellation in the bag with the power morcellator (Figure 3A–D). We have acquired good practical experience with this new system while participating in the ongoing post-market trial sponsored by the manufacturer (Clinicaltrials.gov NCT05537727). We have obtained ethical approval to participate in this study and recruited patients undergoing hysterectomies. The images used in this article are extracted from patients who provided informed consent.

## 7. The Future of Gynecological Surgery

In conclusion, there is an increasing adoption of robotic technology in gynecological surgery worldwide, both in malignant and benign scenarios, particularly in the last five years. Further technical development in RAS together with surgical platforms integrating concurrent technological advancements (such as artificial intelligence) should continue to flatten the learning curve of robotic surgeons by enhancing robotic systems’ intuitive controls, improving real-time feedback and visualization, and providing comprehensive virtual training environments to facilitate skill acquisition. This way, an easier and earlier transition to RAS for surgeons of all experience levels can be provided. Experiencing all the benefits of RAS and expanding its portfolio for complex gynecological surgeries will aid the surgeons’ adoption of robotic technology in the OR. We can anticipate a continued exponential growth in the use of RAS for gynecological procedures in the coming years, along with the implementation of cutting-edge technological advances driven by accelerated research in artificial intelligence.

## Figures and Tables

**Table 1:** Meta-analyses and Randomized Controlled Trials (RCTs) comparing minimally invasive laparoscopic gynecological procedures (LAP) to robotically assisted surgeries (RAS), ordered by level of evidence. OT: Operative Time; LOS: Length of Hospital Stay.

Authors	Disease	Number of Patients (Number of Studies)	Study Design	Study Results
Lenfant et al. [36]	Benign hysterectomy	24 studies	Meta-analysis RCT, prospective and retrospective databases	Overall shorter hospital stay and less blood loss in RAS vs. LAP, no difference in OT
Pickett et al. [35]	Benign Hysterectomy	296 (three studies)	Meta-analysis RCT	Return to normal activities in RAS was lower (low grade evidence and inconclusive results)
Albright et al. [32]	Benign Hysterectomy	326 (four studies)	Meta-analysis RCT	No difference in perioperative complication rates, LOS, OT, conversion, or blood loss
Liu et al. [13] *	Cervical cancer, hysterectomy	19 studies	Meta-analysis, retrospective comparison of LAP, RAS, and abdominal approaches	Longer or equivalent OT, more blood loss, and shorter hospital stay with RAS
Wang et al. [4]	Uterine fibroids, myomectomy	2852 (20 studies)	Meta-analysis, retrospective comparison of LAP, RAS, and abdominal approaches	Fewer intra-operative complications and laparotomy conversions, lower estimated blood loss, and less post-operative bleeding with RAS
Aarts et al. [15] **	Variable benign gynecological diseases, hysterectomy	5102 (47 studies)	Meta-analysis, retrospective comparison of LAP, RAS abdominal, and vaginal approaches	Longer OT and faster return to normal activities with RAS
Tsakos et al. [37]	Uterine fibroids, myomectomy	53 studies	Meta-analysis, retrospective comparison of RAS, LAP, and abdominal approaches	Longer OT, lower blood loss with RAS; equivalent length of stay, transfusion, and complication rates
Narducci et al. [38]	Gynecologic cancer	369	RCT, prospective comparison of LAP and RAS	Longer OT and higher blood loss in RAS, similar conversion rates to open and perioperative morbidity
Lönnerfors et al. [39]	Variable benign gynecological diseases, hysterectomy	122	RCT, prospective comparison of LAP, RAS, and vaginal approaches	Shorter OT, lower blood loss, fewer intra- and post-operative complications
Mäenpää et al. [19]	Endometrial cancer, hysterectomy	99	RCT, prospective comparison of LAP and RAS	Shorter OT with RAS; laparotomy conversion rate higher with LAP
Soto et al. [18]	Endometriosis, endometriosis surgery	73	RCT, prospective comparison of LAP and RAS	Longer OT with RAS; equivalent blood loss, complication rates, and laparotomy conversion rates
Anger et al. [16]	Pelvic organ prolapse, sacrocolpopexy	78	RCT, prospective comparison of LAP and RAS	More postoperative pain with RAS; equivalent complication rates and short-term outcomes
Restaino et al. [17]	Endometriosis, endometriosis surgery	1527	RCT, retrospective comparison of LAP and RAS	Longer OT with RAS; equivalent blood loss, complication rates, and length of hospital stay
Swenson et al. [14]	Variable benign gynecological diseases, hysterectomy	1338	RCT, retrospective comparison of RAS, LAP, and vaginal approaches	Longer OT, lower blood loss, and shorter hospital stays with RAS; equivalent intra- and major postoperative complication rates
Kenton et al. [40]	Pelvic organ prolapse, sacrocolpopexy	78	RCT, retrospective comparison of LAP and RAS	Equivalent one-year follow-up outcomes, return to normal activities, and recurrence rates

* Authors report possible bias due to poor study quality. ** Authors report limitations due to poor reporting and imprecision.

**Table 2:** Technical specifications of the Dexter robotic system™.

Property	Dexter Characteristics
Manufacturer	Distalmotion SA, Switzerland
Robotic system name	Dexter
Approach	Laparoscopic
Clinical approval	European CE Mark (2022)
Patient cart	Two carts with instrument arms, one optional endoscope cart
Arm configuration	Modular/LAP mode functionality
Surgeon console	Open/sterile/ergonomic
Endoscope	Compatible with any 3D endoscope/Indocyanine green florescence imaging system
Endoscope arm	Mountable on the patient bed or endoscope cart
Imaging platform	Compatible with any 3D imaging system installed in front of the surgeon console
Trocars	Compatible with any 10–12 mm trocars
Instruments	Five single-use instruments: needle holder, monopolar hook, monopolar scissors, bipolar Maryland dissector, and bipolar Johann grasper
Instrument diameter/Degree of Freedom	8.3 mm/7°
Foot pedal control	Yes, clutch and endoscope
Simulator available	Yes
Fields of application	Gynecology, general surgery, and urology
Additional features	Easy switching between conventional and robotically assisted laparoscopy thanks to the LAP button on each instrument arm that folds the robot arm without undocking The arm configurations leave a spacious working area around the surgical field and patient bed The robotic system can be integrated with already available Operating Room equipment The surgeon can remain sterile throughout the procedure and move from the console to the patient bed within seconds

## Data Availability

Data are contained within the article.
